# Size and shape matter for micellar catalysis using light-responsive azobenzene surfactants†

**DOI:** 10.1039/d4ob01587h

**Published:** 2024-11-07

**Authors:** Camille Blayo, Beatrice E. Jones, Michael J. Bennison, Rachel C. Evans

**Affiliations:** a School of Chemistry, Trinity College Dublin, College Green Dublin 2 Ireland; b Department of Material Science and Metallurgy, University of Cambridge 27 Charles Babbage Road Cambridge CB3 0FS UK rce26@cam.ac.uk

## Abstract

The micellar catalysis of a model Claisen–Schmidt aldol condensation reaction using heterogeneous nanoreactors based on cationic azobenzene trimethylammonium bromide (AzoTAB) photosurfactants is investigated. Under UV irradiation, AzoTABs undergo a *trans*–*cis* photoisomerisation, which changes not only the critical micelle concentration, but also the shape and size of the micelle. The effect of surfactant structure (tail and spacer lengths), concentration and temperature on the reaction yield were investigated. Monitoring of the zeta potential during the reaction indicated that it proceeds at the micelle/water interface for AzoTABs, with the enolate intermediate stabilised in micelle/water interface (*i.e.* the Stern layer). The reaction yield was found to correlate directly to micellar shape and size, with smaller, more spherical micelles typical of *cis*-AzoTABs favouring higher reaction efficiencies.

## Abstract 2

We show that the reaction conversion efficiency of a Claisen–Schmidt aldol condensation under micellar conditions in water is controlled by the shape and size of the micelle nanoreactors.

## Graphical abstract

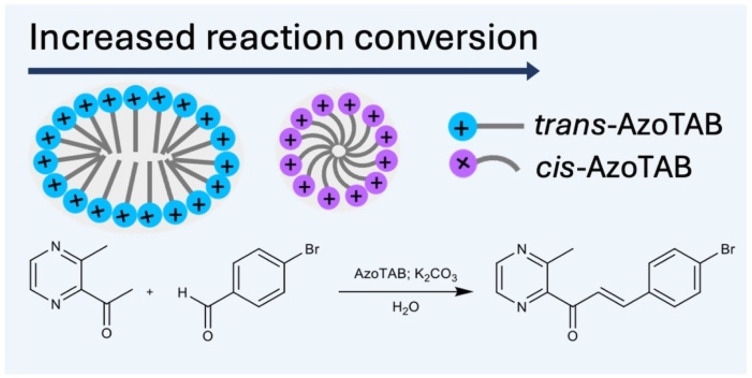

## Introduction

Micellar catalysis is an attractive approach for performing organic chemical reactions in water.^1–3^ Micelles can be envisaged as heterogeneous nanoreactors, comprising a hydrophobic core and hydrophilic surface, dispersed within an apparent homogenous aqueous phase.^4^ Both organic reagents and catalysts can be solubilised in micelles due to intermolecular interactions such as hydrophobic effects^1,5^ and ion pairing.^6^ Moreover, physical confinement of the reaction medium can lead to increased reaction rates, high yields, reduced side reactions and improved selectivity over conventional methods,^1,7^ and by enabling reactions to be performed in water, lead to a reduction of organic solvent waste. Product release is typically achieved by disrupting the micellar structure, for example by reducing the temperature or decreasing concentration.^2,3^

Light-responsive azobenzene (Azo) surfactants have gained increasing attention for micellar catalysis, as both promoters^8–11^ and reagents,^12–14^ as they enable the possibility of on-demand product recovery using light.^15,16^ Azobenzene undergoes *trans* (*E*) to *cis* (*Z*) isomerisation under UV irradiation, forming a photostationary state (PSS) that can be reversed using blue light or heat. Photoisomerisation leads to a substantial change in the geometry, hydrophilicity and packing of azobenzene photosurfactants at the molecular, micellar and mesophase levels,^17–19^ with the critical micelle concentration (CMC) determined by the predominant PSS state. The *cis*-CMC is usually greater than the *trans*-CMC, with a large difference (*i.e.* ΔCMC) favouring higher reaction rates and yields of product recovery.^14^

However, choosing a suitable (photo)surfactant for micellar catalysis is non-trivial. Some reactions can be aided by a certain type of surfactant (*i.e.* anionic, cationic or neutral), while others fail to promote the reaction.^20,21^ This is often due to the location where the reaction is taking place and/or the ability of the micelle to stabilise intermediates.^22^ For example, with neutral surfactants, reactions are typically confined to the hydrophobic micellar core, whereas for ionic surfactants, they are often mediated at the micelle-water interface.^7^ It has also been shown that the relative hydrophobic tail-to-polar head group volume can control the effective loading of reagents in the micellar core or Stern layers.^7^

**Fig. 1:**
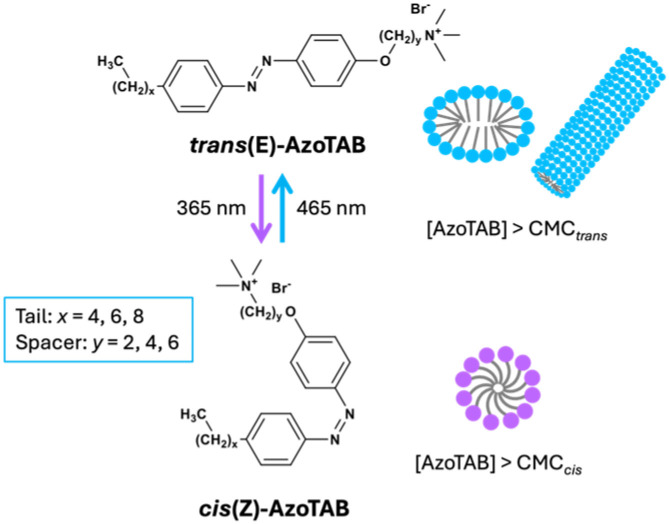
Molecular structure of the AzoTAB photosurfactants investigated. Above the critical micelle concentration (CMC), AzoTABs in the *trans*-dominant photostationary state self-assemble into ellipsoidal or cylindrical micelles (illustrated in blue) depending on the length of the tail (*x*) and alkoxy spacer (*y*) groups. *cis*-AzoTABs tend to form smaller, spherical micelles (purple).

The size and shape of the micellar nanoreactor have empirically been shown to affect the reaction conversion.^3^ However, it is challenging to isolate the role of these factors as they are highly dependent on the surfactant structure. Herein, we exploit the unique ability of photosurfactants to change their micelle shape and size in response to light to directly probe the effect of these factors.^23,24^ As proof-of-concept we examine a representative Claisen–Schmidt aldol condensation reaction performed in micellar nanoreactors constructed from self-assembled cationic azobenzene photosurfactants with a trimethylammonium bromide headgroup (AzoTABs, Fig. 1).

AzoTABs have been investigated for diverse applications ranging from DNA compaction^25^ and protein folding,^26^ to droplet manipulation^27^ and enhanced enzyme activity.^28^ However, to the best of our knowledge they have not yet been investigated for use in micellar catalysis. We have previously shown that subtle modifications to the AzoTAB structure (*e.g.*, alkyl tail (*x*) or alkoxy spacer length (*y*)) strongly influence the hydrophobic-hydrophillic balance upon photoisomerisation, leading to large ΔCMCs and diverse micelle shapes and sizes for each photoisomer (see Table 1 and Tables S1, S2, ESI†).^23,24^ The AzoTAB family thus provides a unique model system to systematically examine the relationship between molecular structure, and how this relates to micelle shape and size, on the catalysis efficiency.

**Table 1:** Effect of surfactant structure, micelle shape and size on the conversion efficiency of the Claisen–Schmidt aldol condensation under micellar conditions

Surfactant	Isomer	Micelle shape^b^ (size)	Yield^c^ (%)
C4AzoOC4TAB	*trans*–*cis*	Oblate ellipsoid (4.1 nm)	46
(*x* = 4, *y* = 4)		Sphere (2.6 nm)	70
*l* = 1.8 nm (*trans*)^a^			
C4AzoOC6TAB	*trans*–*cis*	Oblate ellipsoid (5.0 nm)	48
(*x* = 4, *y* = 6)		Sphere (2.6 nm)	60
*l* = 1.8 nm (*trans*)^a^			
C6AzoOC4TAB	*trans*–*cis*	Ellipsoidal cylinder (16 nm)	64
(*x* = 6, *y* = 4)		Ellipsoidal cylinder (26 nm)	48
*l* = 2.1 nm (*trans*)^a^			
C8AzoOC2TAB	*trans*–*cis*	Oblate ellipsoid (5.3 nm)	53
(*x* = 8, *y* = 2)		Sphere (2.5 nm)	78
*l* = 2.3 nm (*trans*)^a^			
C8AzoOC6TAB	*trans*–*cis*	Oblate ellipsoid (8.7 nm)	55
(*x* = 8, *y* = 6)		Sphere/ellipse (3.6 nm)	62
*l* = 2.3 nm (*trans*)^a^			
CTAB	N/A	Oblate ellipsoid (∼3.2 nm)^d^	66
*l* = 2.2 nm^a^			

a Estimated tail length, *l*, estimated using the Tanford equation^30^ for the alkyl component and the calculated length of the *trans*-azobenzene core^32^ – see Section 3, ESI† for details.

b Determined from small-angle neutron scattering (see ref. 23). Size refers to the longest axis in the micelle shape (see Table S2† for further details).

c Reaction yield from ^1^H NMR (after 2.5 h), *T* = 35 °C, [AzoTAB] ≫ CMC (see Tables S3–S5† for further details).

d From ref. 24.

## Results and discussion

### Effect of reaction conditions

**Fig. 2:**
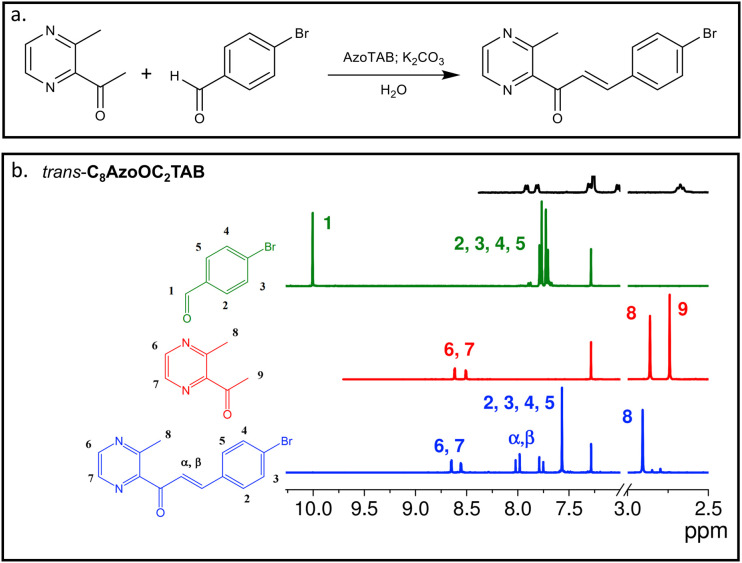
(a) General reaction conditions for the Claisen–Schmidt aldol condensation under micellar conditions. (b) ^1^H NMR spectra in CDCl_3_ of 4-bromobenzaldehyde (green line), 2-acetyl-3-methylpyrazine (red line) and (*E*)-3-(4-bromophenyl)-1-(3-methylpyrazin-2-yl)prop-2-en-1-one (blue line), zoomed in the 7–10 ppm and 2.5–3.0 ppm regions. The conversion of the reaction is given by the disappearance of the characteristic proton peaks 1 and 9, and the appearance of the α,β peaks at 8.0 ppm. The ^1^H NMR spectrum (in CDCl_3_) of *trans*-C_8_AzoOC_2_TAB (top black line) is shown for comparison and to highlight that the AzoTAB is not found in the ^1^H NMR spectrum of the final product.

Previous studies have shown that Claisen–Schmidt aldol condensation of 2-acetyl-3-methylpyrazine and 4-bromobenzaldehyde to form the ((*E*)-3-(4-bromophenyl)-1-(3-methylpyrazin-2-yl)prop-2-en-1-one) α,β-unsaturated ketone (Fig. 2a) can proceed in high yield under aqueous micellar conditions using non-light-responsive cationic quaternary ammonium surfactants.^29^ While dodecyltrimethylammonium bromide (DTAB) was shown to be the most efficient catalyst, competitive results were also reported for cetyltrimethylammonium bromide (CTAB) (89% *vs.* 82%, respectively) under identical reaction conditions (20 mol%) surfactant relative to the reagents, basic conditions at 35 °C for 2.5 h.^29^ We opted to use CTAB as the benchmark non-light-responsive surfactant in this study as the calculated tail length (∼2.2 nm *vs.* 1.7 nm for DTAB, from Tanford's equation^30^) is a better match to the AzoTAB structures under investigation (Table 1).

We first studied the effect of surfactant concentration on the reaction efficiency in water, using *trans*-C_8_AzoOC_2_TAB as a model AzoTAB and CTAB as a control (Table S3, ESI†). C_8_AzoOC_2_TAB was chosen because it exhibits a large ΔCMC (2.4 mM at 20 °C).^23,24^ The reaction conversion efficiency was monitored by ^1^H nuclear magnetic resonance (NMR) spectroscopy of the reaction mixture after filtration, with complete conversion indicated by the loss of both the aldehyde and methyl ketone signals present in the reagents, and the appearance of the α,β-unsaturated alkene of the product (Fig. 2b). The highest conversion (53%) was obtained for 13.6 mM (15 mol%) C_8_AzoOC_2_TAB in water at 35 °C, which is competitive to the yield reported for CTAB (66%) under identical conditions.^29^ Moreover, the lower CMC of *trans*-C_8_AzoOC_2_TAB meant that a comparable reaction yield could also be obtained using a ten-fold lower surfactant concentration (0.5 mM (2 mol%), yield ∼ 52%). In contrast, the absence of micelles resulted in negligible reaction for CTAB at this concentration (CMC∼1 mM in water^31^).

The effect of temperature was also examined (see Table S4, ESI†). At 20 °C, significantly lower reaction conversions were obtained: 14% for CTAB (20 mol%) and 19% for *trans*-C_8_AzoOC_2_TAB (16 mol%), respectively. This is unsurprising since this temperature is below the Krafft point of both surfactants (*T*_Krafft_ (CTAB) = 25 °C,^33^*T*_Krafft_ (*trans*-C_8_AzoOC_2_TAB) = 23 °C). Notably, for *trans*-C_8_AzoOC_2_TAB, increasing the temperature to 70 °C did not significantly increase the reaction conversion compared to 35 °C (56% *cf*. 53%, respectively, see Table S4, ESI†). This highlights the advantage of using micellar catalysis to perform the Claisen–Schmidt aldol condensation under relatively mild conditions compared to traditional synthetic processes requiring strong base/acid or expensive catalysts^34,35^ in organic solvents which require specific conditions (temperature, inert atmosphere).^36^

### Where does the reaction take place?

**Fig. 3:**
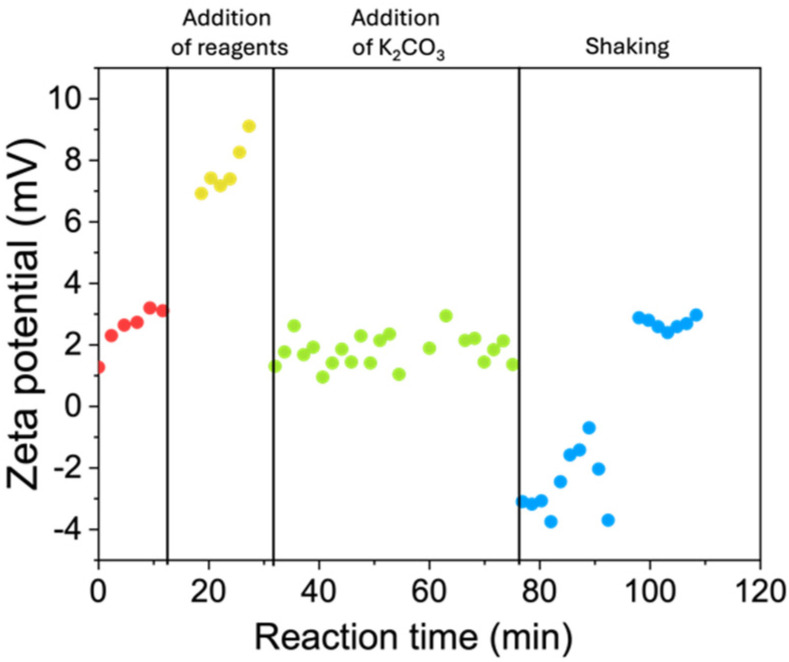
Evolution of the zeta potential during the reaction between 4-bromoaldehyde and 2-acetyl-3-methylpyrazine, promoted by *trans*-C_8_AzoOC_2_TAB at 10 mM in water and 35 °C, as a function of time. The solid black lines correspond to visual indication of the addition of reagents to the micellar solution, K_2_CO_3_ and after shaking and are followed by abrupt changes to the zeta potential.

For micellar catalysis promoted by cationic surfactants, the location of the reaction is a source of debate.^6^ The Claisen–Schmidt aldol condensation reaction using CTAB is purported to occur at the micelle/water interface, with basic conditions promoting the formation of the anionic enolate intermediate that is stabilised in the Stern layer.^29^ To investigate the role of nanoscale electrostatic interactions at the micelle surface on the reaction rate using AzoTABs, the zeta potential (ZP) was monitored as a function of reaction time (Fig. 3). The ZP of *trans*-C_8_AzoOC_2_TAB in micellar solution (10 mM) is +2.5 ± 0.7 mV, which is as expected for a highly concentrated solution of cationic micelles. Upon addition of the reagents (*t* = 10 min), but in the absence of base which is required for the reaction to proceed, a sharp increase in the ZP to +7.7 ± 0.8 mV is observed, which continues to increase over ∼10 min +9.1 mV. This result suggests that the reagents interact with the surface of the cationic micelles. Following addition of K_2_CO_3_ to initiate the reaction (*t* = 20 min), a sudden decrease in surface charge to +2.0 ± 0.7 mV was observed. The positive ZP was initially surprising as the negatively-charged enolate intermediate was expected to form. However, after a period of diffusion-controlled mixing (15 min), followed by manual shaking (2 min), the ZP dropped to −3.1 mV supporting enolate formation. As the reaction progressed, the ZP gradually increased, indicative of the consumption of the enolate, reaching a plateau at +2.7 ± 0.2 mV after 30 minutes. As the reaction proceeds, a colourless precipitate forms (the α,β-unsaturated ketone product) and the ZP returns to its initial value. We note that in a control experiment replicating this procedure for just *trans*-C_8_AzoOC_2_TAB and K_2_CO_3_ (*i.e.* no reagents added), while the ZP was observed to decrease on K_2_CO_3_ addition, negative ZP values were not obtained after 30 minutes reaction (Fig. S1, ESI†).

### Effect of surfactant structure

Next, the effect of the surfactant structure on the reaction efficiency was investigated at 35 °C, using a surfactant concentration of 10 mM in water (15 mol% ratio relative to the reagents), which is above the CMC for all *trans*-AzoTABs. The reaction conversion efficiencies are summarised in Table 1. Previously, small-angle neutron scattering (SANS) studies have shown that AzoTABs form elongated micelles of different shapes, sizes and aggregation numbers in this concentration regime in deuterated aqueous solutions (see Table 1 and Table S2, ESI†).^23,24^ A direct relationship between the size and elongation of micelles formed and the conversion to product was observed. For *trans*-AzoTABs forming oblate ellipsoidal micelles (all except C_6_AzoOC_4_TAB), the long axis of the micelle generally increases with the combined length of the alkyl tail and alkoxy spacer (*i.e.*, *x* + *y*) and leads to a corresponding increase in the reaction conversion efficiency. This observation suggests that there is a direct relationship between the micellar volume and the reaction efficiency. We note that both the CMC and micelle shape and volume will be affected by the medium and so this correlation to the reaction efficiency can only be directly applied to the performance in water.

Our results show that the Claisen–Schmidt aldol condensation is catalysed by *trans*-AzoTABs under micellar conditions, with the cationic Stern layer supporting the formation of the enolate intermediate. Moreover, reaction efficiencies that are comparable to CTAB can be achieved for some AzoTAB structures. However, it is as yet unclear whether the observed differences in reaction conversion are due to the change in molecular structure and micelles size alone, or whether the micelle shape is also important. With this in mind, we next investigated micellar solutions prepared from *cis*-AzoTABs, in which aqueous solutions of the surfactant were pre-irradiated with UV light (365 nm, 10 min). The surfactant concentration was 10 mM (*i.e.* above CMC_*cis*_ for all AzoTABs, Table S1, ESI†) and formation of the *cis*-PSS was confirmed by UV/Vis absorbance spectroscopy (Fig. S1, ESI†). At this concentration, all *cis*-micelles are expected to be spherical and similar in size (see Table 1), with a short interparticle distance and a small aggregation number (again, except for *cis*-C_6_AzoOC_4_TAB, see Table S2†).^19^ It is worth noting that spherical cationic micelles are not commonly reported in the literature, as micelles are often cylindrical or ellipsoidal.^37^ For all *cis*-AzoTABs that form spherical micelles, the reaction conversion efficiency increased significantly (1.1–1.5×) compared to their *trans*-counterparts under analogous reaction conditions. *cis*-C_8_AzoOC_2_TAB showed the highest reaction conversion of all surfactants studied (78%), including the CTAB control. C_6_AzoOC_4_TAB is anomalous as it forms ellipsoidal cylinders for both photoisomers, with the *trans*-PSS forming smaller micelles than the *cis*-PSS. Accordingly, the reaction conversion efficiency is reversed for this AzoTAB, *i.e. trans* > *cis* (Table 1). We attribute the higher reaction yields generally obtained for *cis*-AzoTABs to the thicker shell present in the spherical micellar aggregates, that is better able to stabilise the enolate intermediates and thus promote the reaction. Moreover, the smaller average micelle size (and thus volume) in the *cis*-PSS should decrease the mean distance between the enolate intermediate (in the Stern layer) and the aldehyde reactant (solubilised in the hydrophobic micelle core), further increasing the reaction probability. This rationale is supported by the small efficiency increase observed for *cis*-C_8_AzoOC_6_TAB (1.1×) which forms a slightly larger spherical micelle, with some eccentricity.^23,24^

### Recyclability

The potential recyclability of the AzoTAB micellar solution for re-use in subsequent reactions was investigated by directly taking the reaction filtrates containing either *trans*-C_8_AzoOC_2_TAB and *trans*-C_6_AzoOC_4_TAB after filtration, but before the precipitated product was washed with water. The AzoTAB filtrate showed a 5–10% reduction in concentration (determined by UV/Vis absorbance spectroscopy), but remained higher than the CMC for both samples. A repeat reaction performed with a recycled solution of *trans*-C_6_AzoOC_4_TAB gave a lower conversion (48%), with a subsequent third run again leading to a further reduction in conversion (35%). In comparison, the recovered *trans*-C_8_AzoOC_2_TAB solution could not be recycled, with no reaction observed. This could possibly indicate that the micelle size and shape are affected by the presence of unreacted reagents, which modify the accessibility of the cationic charge at the micelle surface to form the enolate, inhibiting the reaction.

Finally, we explored whether the photoresponse of AzoTABs could be used to enhance product recuperation. *In situ* UV irradiation was performed at the end of the reaction using *trans*-C_8_AzoOC_2_TAB (0.5 mM in water). At this concentration (*i.e.* below CMC_*cis*_), conversion to the *cis*-PSS was expected to result in disruption of the micelle structure and facilitate product recuperation. However, surprisingly this treatment led to significantly lower product conversion (31% and 52%, respectively by ^1^H NMR, see Table S6, ESI†) and lower recovery yields compare to a non-irradiated sample. We believe this could be due to undesired photodegradation of the α,β-unsaturated ketone product.^34^

## Conclusions

In summary, we have demonstrated that AzoTABs can be successfully employed in the micellar catalysis of the Claisen–Schmidt aldol condensation in water at 35 °C. The reaction conversions obtained by ^1^H NMR are comparable to those obtained using a CTAB control, with the added advantage that decreased surfactant loadings could be used due to the lower CMCs of the AzoTABs. Monitoring of the zeta potential during the reaction indicated that it proceeds at the micelle/water interface for AzoTABs, with the enolate intermediate stabilised in the Stern layer. Notably, the reaction efficiency could be tuned by small changes to the molecular structure of the AzoTAB and the photoisomer present, which have a direct effect on the size and shape of the micelles formed. The reaction efficiency was found to correlate directly to the relative micelle volume to micelle/water interface area (*i.e.*, the Stern layer), with smaller, more spherical micelles typical of *cis*-AzoTABs favouring higher reaction efficiencies. This result highlights not only the importance of considering the effect of molecular structure on the shape and size of the micelles formed, but also how this relates to the interfacial area where any reactive intermediates are stabilised. As the nature (*i.e.* pH, ionic strength) of the bulk medium will also determine the CMC of the surfactant and micellar shape and volume, future studies should examine effect this may have on the reaction efficiency.

While the photoresponse of the AzoTABs could not be harnessed to increase product recuperation for this reaction due to photodegradation of the product, we anticipate that with appropriate choice of reaction this should be achievable. Our aim is that judicious application of the insight gleaned here will stimulate the strategic design and application of new photoswitchable surfactants for micellar catalysis of diverse reaction schemes.

## Experimental

### Materials

The synthesis and structural characterisation of all cationic alkylazobenzene trimethylammonium bromide (AzoTAB) photosurfactants used in this study were previously reported.^23^ AzoTAB structures are designated as C_*x*_AzoOC_*y*_TAB, where *x* denotes the number of carbon atoms in the alkyl tail and *y* refers to the number of atoms in the alkoxy spacer group that separates the azobenzene core from the trimethylammonium bromide head group (see Fig. 1). Full details of the reagents used in the aldol condensation reaction can be found in the ESI (section 1†).

### Claisen–Schmidt aldol condensation under micellar conditions

In a typical experiment, 4-bromobenzaldehyde (63.4 mg, 0.34 mmol) was weighed in a round-bottom flask (10 mL) and Millipore™ water was added (5 mL). The solution was stirred at room temperature and 2-acetyl-3-methylpyrazine (40.5 μL, 0.34 mmol) was added. To this solution, the photosurfactant *trans*-**C**_**8**_**AzoOC**_**2**_**TAB** (25 mg, 0.03 mmol) was added, followed by K_2_CO_3_ (46.2 mg, 0.35 mmol). The solution was heated to 35 °C, protected from external light by tin foil and stirred for 2.5 h. The reaction was followed by thin layer chromatography (cyclohexane : ethyl acetate; 9 : 1), cooled to 4 °C for 30 min and filtered. The filtrate product was collected, washed with Millipore™ water (15 mL) and dried to afford a pale yellow-green powder with a 67% crude mass recovery. The product was recrystallised from ethanol : methanol (9 : 1) to afford the pure (*E*)-3-(4-bromophenyl)-1-(3-methylpyrazin-2-yl)prop-2-en-1-one product, which was characterised by nuclear magnetic resonance spectroscopy (NMR, ^1^H, ^13^C) and high-resolution mass spectrometry (HRMS).

^1^H NMR (CDCl_3_, 400 MHz, 25 °C): *δ* = 2.89 (s, 3H, H_8_), 7.54 (s, 4H, H_2,3,4,5_), 7.75–7.79 (d, *J* = 15.0 Hz, 1H, H_α_), 7.98–8.02 (d, *J* = 15.0 Hz, 1H, H_β_), 8.53 (d, *J* = 2.0 Hz, 1H, H_7_), 8.63 (d, *J* = 2.0 Hz, 1H, H_6_) ppm.

^13^C NMR (CDCl_3_, 100 MHz, 25 °C): *δ* = 23.2, 123.2, 124.9, 130.0, 132.1, 133.6, 140.6, 143.5, 145.6, 147.5, 155.1, 190.5 ppm.

HRMS (CHCl_3_, *m*/*z*-ESI+): found: 303.0125 [M + H]^+^. Calculated: 303.0128 [M + H]^+^.

Comparable conditions were used for other AzoTABs, with the AzoTAB concentration adjusted as required to ensure that it was greater than the critical micelle concentration of the photoisomer being examined. The concentration of surfactant is expressed in terms of both relative concentration (mol%) and absolute concentration (mM). The relative concentration corresponds to the molar ratio of surfactants relative to the reagents added. Literature reports state that a minimum of 1 mol% surfactant is required to catalyse the reaction, and 10–20 mol% is not unusual.^6,7^ However, the absolute concentration is more relevant for surfactants as it provides an indication of the concentration relative to the critical micelle concentration (CMC), *i.e.*, it indicates whether the system is in the micellar state at a given concentration.

### Determination of reaction yields

The reaction yield was determined by ^1^H NMR spectroscopy on a Bruker DPX AC400 instrument at 20 °C using an operating frequency of 400 MHz. Chemical shifts were calibrated against a tetramethylsilane (TMS) signal and are reported in parts per million (ppm). After a fixed reaction time (2.5 h), a small amount of product (5 mg) was dissolved in CDCl_3_ for NMR analysis and used to calculate the reaction yield by comparison of the integral ratios of key signals from starting materials and product. For 4-bromobenzaldehyde, the aldehyde proton was used (*δ*H = 9.9 ppm, 1H, singlet), while the methyl ketone signal (*δ*H = 2.7 ppm, 3H, triplet) was used for 2-acetyla-3-methylpyrazine. The product, (*E*)-3-(4-bromophenyl)-1-(3-methylpyrazin-2-yl)prop-2-en-1-one, was characterised by the geminal protons across the alkene (*δ*H = 7.8 ppm, 4H, quartet of doublets).

### Zeta potential study

The ZP as a function of reaction time was determined from the electrophoretic mobility of micelles using a Zetasizer Nano series nano-ZS. 4-Bromobenzaldehyde (18.7 mg, 0.10 mmol) was dissolved in a solution of *trans*-C_8_AzoOC_2_TAB (10.5 mM, 2 mL) in Millipore™ water. 2-acetyl-3-methylpyrazine (11.9 μL, 0.10 mmol) was added to the solution and left to stir at 35 °C for 5 minutes. K_2_CO_3_ (16.0 mg, 0.13 mmol) was added to the mixture at 35 °C. An aliquot (750 μL) of either *trans*-C_8_AzoOC_2_TAB or *trans*-C_8_AzoOC_2_TAB containing the reagent solutions was added to a folded capillary cell and the ZP was measured at 35 °C. Each measurement was run 6 times. After addition of K_2_CO_3_, an aliquot (750 μL) of the reaction mixture was added to a folded capillary cell and the ZP was monitored as a function of time. Each measurement consisted of 15 runs of 10 s each. The change of surface charge was followed *in situ*. The reaction was run for 110 minutes. An additional control experiment of *trans*-C_8_AzoOC_2_TAB (10.5 mM, 2 mL), followed by K_2_CO_3_ (16.0 mg, 0.13 mmol) addition (but no reagents) was also monitored under the same conditions (Fig. S1, ESI†).

### Photoirradiation conditions

Photoconversion of native *trans*-surfactants to the *cis*-photostationary state was obtained by exposure of solutions to a UV light-emitting diode (LedEngin®) with an illumination wavelength of 365 nm and a power output of 5 mW cm^−2^, when placed at 6 cm from the sample. Conversion from the *trans*-PSS to the *cis*-PSS was confirmed by UV/Vis absorbance spectroscopy (Fig. S2, ESI†). An exposure time of 10 minutes was found to be sufficient to generate the *cis*-PSS and was used unless otherwise stated.

### Recyclability studies

The recyclability of micellar solutions prepared from *trans*-**C8AzoOC2TAB** and *trans*-**C6AzoOC4TAB** was investigated. After completion of the first reaction, the mixture was filtered and the solid powder product was collected to perform NMR analysis and calculate the yield. The filtrate (≈5 mL) was collected and transferred into a round bottom flask (10 mL). Fresh 4-bromobenzaldehyde and 2-acetyl-3-methylpyrazine in a 1 : 1 molar ratio were added to the filtrate, along with K_2_CO_3_ (1.3 mol eq.). The time and temperature of the recyclability reaction were kept identical to the initial reaction. This process was repeated two times for each surfactant.

## Author contributions

C. B: conceptualisation, methodology, investigation, data curation, formal analysis, writing – original draft. B. E. J.: investigation, data curation, formal analysis, writing – reviewing & editing. M. J. B.: data curation, writing – reviewing & editing. R. C. E.: conceptualisation, project administration, resources, funding acquisition, supervision, writing – reviewing & editing.

## Data availability

The data supporting this article have been included as part of the ESI.†

## Conflicts of interest

There are no conflicts to declare.

## Supplementary Material

OB-023-D4OB01587H-s001
