# Supplementary material for: Size and shape matter for micellar catalysis using light-responsive azobenzene surfactants
Source: Org Biomol Chem. 2024 Nov 7;23(1):138–44. doi: 10.1039/d4ob01587h (PMC11563304; doi:10.1039/d4ob01587h)
Supplement: OB-023-D4OB01587H-s001 [file OB-023-D4OB01587H-s001.pdf]

Supplementary Information (SI) for Organic & Biomolecular Chemistry.
This journal is © The Royal Society of Chemistry 2024

Supporting Information

Size and Shape Matter for Micellar Catalysis using Photosurfactants

Camille Blayo,[a] Beatrice E. Jones,[b] Michael J. Bennison,[b] and Rachel C. Evans[b]*

[a]*School of Chemistry, Trinity College Dublin, Dublin 2, Ireland*

[b]*Department of Materials Science & Metallurgy, University of Cambridge, 27 Charles Babbage Road, Cambridge, CB3 0FS, United Kingdom*

** Corresponding Author: Rachel C. Evans (rce2C@cam.ac.uk)*

TABLE OF CONTENTS

1 Materials 2

2 Supporting Data 2

2.1 Physicochemical and self-assembly properties of AzoTABs 2

2.2 Effect of reaction conditions on conversion efficiency and product yield 4

2.3 Effect of UV irradiation 5

2.4 Supporting Zeta Potential Measurements 6

2.5 UV/Vis Absorption Spectroscopy 6

3 Estimation of surfactant tail length 6

4 References 7

1 Materials

4-bromobenzaldehyde (95%), 2-cetyl-3-methylpyrazine (97%), chloroform and hydrochloric acid (conc. 37%) were purchased from Fisher Scientific, K_2CO_3 (≥99%), cyclohexane, cetyltrimethyl ammonium bromide (CTAB, 95%) and ethanol were purchased from Sigma Aldrich, and phenol (≥99%) was purchased from BDH Chemical Ltd. $CDCl_3$ (99.8%), DMSO-d6 (99.8%) and D_2O (99.92%) were purchased from Apollo Scientific Limited. All solvents used for reactions were analytical grade and for characterisation were HPLC grade. Millipore^{TM} water was obtained from passing distilled water through a Mill-Q purifier (resistivity = 18.2 MΩ cm^{-1} at 25 °C).

2 Supporting Data

2.1 Physicochemical and self-assembly properties of AzoTABs

Table S1: Critical micelle concentrations (CMCs) for the *trans*- and *cis*-photoisomers of AzoTABs and the Krafft temperature of the trans-form.

**Surfactant**	**CMC*trans* (mM)[a]**	**CMC*cis* (mM)[a]**	**ΔCMC (mM)[b]**
C_4AzoOC_4TAB	1.2 ± 0.1	2.6 ± 0.2	2.4
C_4AzoOC_6TAB	0.4 ± 0.1	1.1 ± 0.1	0.7
C_8AzoOC_2TAB	0.2 ± 0.1	0.7 ± 0.1	0.5
C_8AzoOC_6TAB	0.3 ± 0.1	0.8 ± 0.1	0.5
C_6AzoOC_4TAB	0.1 ± 0.1	0.2 ± 0.1	0.1
CTAB[c]	˜1.0[c]		

[a] Mean value from surface tensiometry and dynamic light scattering measurements. T = 20 °C. Data from reference [1]. [b] ΔCMC = CMC*cis*- CMC*trans* [c] From reference [5].

Table S2. Summary of the shape and size of *trans*- and *cis*-AzoTAB micelles in D_2O at 25 °C as determined from model fitting to small-angle neutron scattering. Data summarised from reference [1] to aid discussion. The reader is referred to the original paper for detailed discussion of the model fitting.

Surfactant	Isomer	Conc. (mM)	Form factor, $P(q)$	R [a] (Å)	L [b] (Å)	H [c] (Å)	N_{agg} [d]
C_4AzoOC_4TAB[b]	*trans*	5	Oblate Ellipsoid[e]	25 ± 3	39 ± 2	-	90 ± 14
		20		24 ± 1	41 ± 1	-	165 ± 10
	cis	5	concentration too close to the CMC to yield useful scattering profile				
		20	Sphere[e]	26 ± 1	-	-	42 ± 8
C_4AzoOC_6TAB	*trans*	5	Oblate Ellipsoid[e]	25 ± 1	45 ± 1	-	119 ± 13
		20		26 ± 1	50 ± 1	-	255 ± 24
	cis	5	Sphere[e]	24 ± 1	-	-	26 ± 5
		20		26 ± 1	-	-	67 ± 10
C_8AzoOC_2TAB	*trans*	5	Oblate Ellipsoid[e]	20 ± 1	52 ± 1	-	209 ± 21
		20		24 ± 1	53 ± 1	-	309 ± 22
	cis	5	Sphere[e]	22 ± 1			47 ± 5
		20		25 ± 1			72 ± 10
C_8AzoOC_6TAB	*trans*	5	Oblate Ellipsoid[e]	28 ± 1	88 ± 1	-	669 ± 30
		20		29 ± 1	87 ± 1	-	753 ± 27
	cis	5	Sphere[e]	28 ± 1			64 ± 8
		20	Oblate Ellipsoid[e]	23 ± 1	36 ± 1		140 ± 13
C_6AzoOC_4TAB	*trans*	5	Elliptical cylinder[e]	28 ± 1	49 ± 1	364 ± 11	-
		20		27 ± 1	53 ± 1	162 ± 2	-
	cis	20	Elliptical cylinder[e]	22 ± 1	46 ± 1	260 ± 9	572 ± 25

[a] Polar radius. [b] Equatorial radius [c] Cylinder length [d] N_{agg} is the aggregation number for the micelle. [e] Hayter-Penfold structure factor for electrostatic interactions included in this fit as described in reference [1].

2.2 Effect of reaction conditions on conversion efficiency and product yield

Table S3: Effect of surfactant concentration in water on the reaction yield (after 2.5 h) for the Claisen-Schmidt aldol condensation under micellar conditions at T = 35 °C.

Surfactant	(mM)	(mol %)[c]	Base [d]	^{1}H NMR (%)
CTAB[a]	13.5	15	K_2CO_3	66
CTAB	0.5	2	K_2CO_3	trace
trans-C_8AzoOC_2TAB[b]	13.6	15	K_2CO_3	53
trans-C_8AzoOC_2TAB	0.5	2	K_2CO_3	52
trans-C_8AzoOC_2TAB	13.6	15	None	trace
None	-	-	K_2CO_3	24

[a] CMC = 0.01 mol %/ 1 mM in water; [b]CMC = 0.005 wt%/ 0.5 mM in water [c] Relative concentration is the mol% of surfactant relative to the reagents. [d] K_2CO_3 is used as base to promote the reaction.

Table S4: Effect of temperature on the reaction yield (after 2.5 h) for the Claisen-Schmidt aldol condensation under micellar conditions in water.

Surfactant	Temperature (°C)	Relative Conc. (mol %)[a]	Reaction Yield ^{1}H NMR (%)
CTAB	20	20	14
trans-C_8AzoOC_2TAB	20	16	19
CTAB	35	13.5	66
trans-C_8AzoOC_2TAB	35	13.6	53
trans-C_8AzoOC_2TAB	70	10.5	56

[a] Relative concentration is the mol% of surfactant relative to the reagents.

Table S5: Effect of the AzoTAB structure on the reaction yield (after 2.5 h) of the Claisen-Schmidt aldol condensation under micellar conditions at T = 35 °C. K_2CO_3 is added as a base to promote the reaction.

AzoTAB	Conc. (mM)[a]	Relative Conc. (mol %)[b]	Reaction Yield ^1H NMR (%)
trans-C_4AzoOC_4TAB	10.0	13	46
trans-C_4AzoOC_6TAB	11.1	13	48
trans-C_8AzoOC_2TAB	13.6	15	53
trans-C_8AzoOC_6TAB	10.5	13	55
trans-C_6AzoOC_4TAB	10.5	15	64

[a] All AzoTAB concentrations are above the CMC at T = 35°C. [b] Relative concentration is the mol% of surfactant relative to the reagents. [c]

2.3 Effect of UV irradiation

Table S6: Effect of UV irradiation on the on the reaction yield (after 2.5 h) of the Claisen-Schmidt aldol condensation under micellar conditions at T = 35 °C. K_2CO_3 (1.3 mol eq. relative to reagents) is added as a base to promote the reaction in all cases.

Surfactant	Conc. (mM)	Relative Conc. (mol %)[c]	Reaction Yield ^1H NMR (%)
cis-C_4AzoOC_4TAB[a]	10.5	13	70
cis-C_4AzoOC_6TAB[a]	10.5	15	60
cis-C_6AzoOC_4TAB[a]	10.5	13	48
cis-C_8AzoOC_2TAB[a]	10.5	15	78
cis-C_8AzoOC_6TAB[a]	10.5	13	62
trans-C_8AzoOC_2TAB	0.5	2	52
trans-C_8AzoOC_2TAB	0.5[b]	2	31

[a] The solution was irradiated at λ_{ex} = 365 nm for 10 minutes prior to the addition of reagents and the start of the reaction. Formation of the *cis*-photostationary state was confirmed by UV/Vis absorbance spectroscopy. [b] The concentration is below the CMC of the *cis*-isomer. The solution was irradiated at λ_{ex} = 365 nm for 10 minutes at the end of the reaction. [c] Relative concentration is the mol% of surfactant relative to the reagents.

2.4 Supporting Zeta Potential Measurements

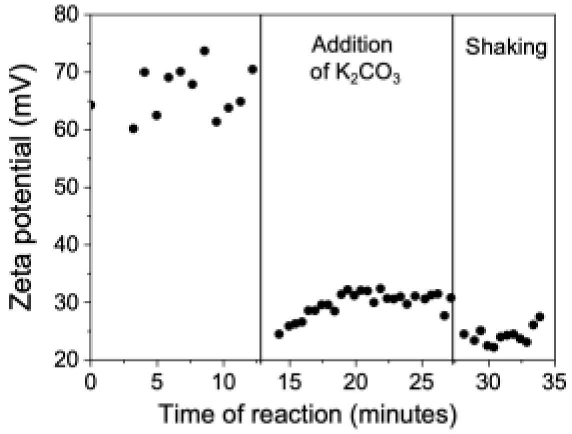

Figure S1: Zeta potential (ZP) as a function of time for ***trans*-C_8AzoOC$_2$TAB** (10 mM in water, 35°C). The solid black lines indicate the addition of K_2CO_3 to the solution, followed by shaking. A negative ZP is not obtained.

2.5 UV/Vis Absorption Spectroscopy

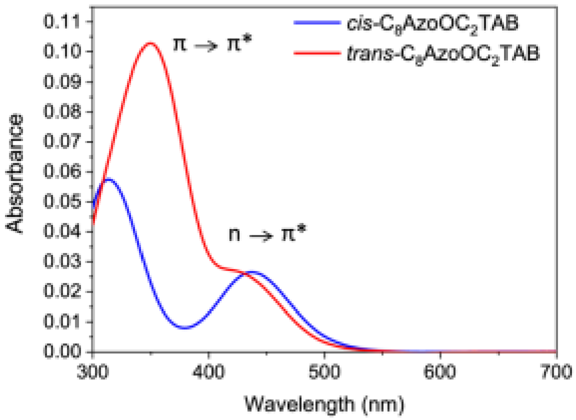

Figure S2: UV/Vis absorbance spectra of ***trans*-C8AzoOC2TAB** (0.1 μmol dm^{-3}, red line) and ***cis*-C8AzoOC2TAB** (0.1 μmol dm^{-3}, blue line) in water. A sample of the reaction mixture (10 μL) was taken and diluted in water (3,000 μL) to evaluate the concentration of AzoTAB from the molar absorption coefficient (≈2.0 × 10^{4} mol^{-1} cm^{-1}) of the *trans*-isomer at λ_{abs} = 350 nm.

3 Estimation of surfactant tail length

The length of the hydrophobic tails, l, of each surfactant were estimated using the empirical volume additivity rule of Traube[6]:

[illegible] Eq. S1

where l_i is the contributions of the *i*th component for the tail length, respectively, using the data from Table S7.

Table S7: Contribution of lengths used to estimate the hydrophobic tail length of different surfactants in the *trans (E)* isomer.

Component	Value	Method
Alkyl chain volume (nm^3)	0.0269*m* + 0.0274	Tanford equations [7]
Z-azobenzene length (nm)	0.9	X-ray analysis[8]
Oxy-group length (nm)	0.28	DFT calculations[9]

4 References

1. C. Blayo, J. E. Houston, S. M. King, R. C. Evans, Unlocking Structure–Self-Assembly Relationships in Cationic Azobenzene Photosurfactants. *Langmuir* **2018**, 34, 34, 10123–10134.
2. B. S. Kitawat, M. Singh, R. K. Kale, Robust Cationic Quaternary Ammonium Surfactant-Catalyzed Condensation Reaction for (E)-3-Aryl-1-(3-alkyl-2-pyrazinyl)-2-propenone Synthesis in Water at Room Temperature. *ACS Sustainable Chem. Eng.* **2013**, 1, 8, 1040–1044.
3. T. Kitanosono, K. Masuda, P. Xu, S. Kobayashi, Catalytic Organic Reactions in Water toward Sustainable Society. *Chem. Rev.* **2018**, 118, 679–746.
4. T. Dwars, E. Paetzold, G. Oehme, Reactions in Micellar Systems. *Angew. Chem. Int. Ed.* **2005**, 44, 7174– 7199.
5. M. Bielawska, A. Chodzińska, B. Jańczuk, A. Zdziennicka, Determination of CTAB CMC in Mixed Water + Short-Chain Alcohol Solvent by Surface Tension, Conductivity, Density and Viscosity Measurements. *Colloids Surf. A*, **2013**, 424, 81–88.
6. Durchschlag, H.; Zipper, P. *Calculation of the Partial Volume of Organic Compounds and Polymers*; **1GG4**.
7. Tanford, C. Thermodynamics of Micelle Formation: Prediction of Micelle Size and Size Distribution. *Proc Natl Acad Sci U S A* **1G74**, *71*, 1811–1815.
8. Merino, E.; Ribagorda, M. Control over molecular motion using the cis–trans photoisomerization of the azo group. *Beilstein J. Org. Chem.* **2012**, *8*, 1071-1090.
9. Agapito, F.; Costa Cabral, B. J.; Martinho Simões, J. A. Carbon-Hydrogen Bond Dissociation Enthalpies in Ethers: A Theoretical Study. *Journal of Molecular Structure: THEOCHEM* **2005**, 719, 109–114.
